# An Improved Humidity Sensor with GO-Mn-Doped ZnO Nanocomposite and Dimensional Orchestration of Comb Electrode for Effective Bulk Manufacturing

**DOI:** 10.3390/nano12101659

**Published:** 2022-05-12

**Authors:** Balashanmugam Priyadharshini, Prasad Valsalal

**Affiliations:** Department of Electrical and Electronics Engineering, CEG Campus, Anna University, Chennai 600025, Tamil Nadu, India; valsalal@annauniv.edu

**Keywords:** GO nanocomposite, in-plane humidity sensing, metal-etching, water intercalation

## Abstract

The measurement and control of humidity is a major challenge that affects the sensing properties of sensors used in high-precision equipment manufacturing industries. Graphene Oxide(GO)-based materials have been extensively explored in humidity sensing applications because of their high surface area and functional groups. However, there is a lack of effective bulk-manufacturing processes for the synthesis of 2D-based nanocomposites with comb electrodes. Moreover, water intercalation within the layers of 2D materials increases recovery time. This work demonstrates the enhanced sensing characteristics of a capacitive/resistive GO-MnZnO nanocomposite humidity sensor produced using a cost-effective single-pot synthesis process. The in-plane sensing layer consistently improves sensitivity and reduces response time for a wide range of relative humidity measurements (10% to 90%). Interdigitated gold electrodes with varying numbers of fingers and spacing were fabricated using photolithography on a Si/SiO₂ for a consistent sensor device platform. The choice of nanomaterials, dimension of the sensor, and fabrication method influence the performance of the humidity sensor in a controlled environment. GO nanocomposites show significant improvement in response time (82.67 times greater at 40% RH) and sensitivity (95.7 times more at 60% RH). The response time of 4.5 s and recovery time of 21 s was significantly better for a wider range of relative humidity compared to the reduced GO-sensing layer and ZnMnO. An optimized 6 mm × 3 mm dimension sensor with a 28-fingers comb was fabricated with a metal-etching process. This is one of the most effective methods for bulk manufacturing. The performance of the sensing layer is comparable to established sensing nanomaterials that are currently used in humidity sensors, and hence can be extended for optimal bulk manufacturing with minimum electrochemical treatments.

## 1. Introduction

The research and development of 2D materials in humidity sensing are progressing at such a rapid pace that the limitations in the technology for measuring minute changes in electrical parameters are becoming more significant. Manufacturing more sophisticated, high-precision equipment drastically increases the cost of synthesizing, characterizing, and testing the materials. Even though there are advancements in material science, the challenge to thrive effectively with the established materials is an ever-growing subject of research. One such material to be considered in humidity sensing is graphene, which is very much compared with recently developing Mxene. The need for more precise humidity sensing and control is a rising challenge in most industrial applications. Optimized control of humidity requires versatile sensing characteristics of the humidity-sensing device. The measurement of water vapor in gas, i.e., hygrometry, is comparatively more complex than measuring the temperature of the same environment, though both parameters are related to each other [1]. For example, textile industries require fairly humid conditions to avoid the buildup of dangerous electrostatic charges and maintain the quality of output; whereas dry conditions are necessary for the production of silicon wafers in clean rooms.

Hygrometry is in fact “a branch of applied physics in which the multitude of techniques is an indication of the complexity of the problem, and of the fact that no one solution will meet all requirements at all times and in all places” [2]. Fast response, high sensitivity, quick recovery times, longevity, and bulk production are the significant characteristics to be considered for improving the performance of the sensor. The sensing layer material used for the humidity sensor plays a major role in sensing characteristics. A wide variety of materials, including thin-film 2D materials, polymers, metal oxides, and other composites, have been implemented in humidity sensors [3,4,5,6,7]. Synthesis of thin-film nanomaterial requires complex processes such as sputtering, laser deposition, electrodeposition, etc. [8,9]. These require cleanroom environments that significantly increase manufacturing costs for the bulk production of nanomaterials. Advancements in the nano synthesis of materials have resulted in a drastic improvement in their humidity-sensing properties. However, various experiments need to be done to improve the long-term stability of the sensing nano-materials [10,11]. It is found that the electrical properties of various sensor materials change with doping concentrations, film thickness, and morphological structure of the materials [12,13]. Flexible humidity sensors are still under development in advancement for wearable applications, and yet accurate measurements due to bending are affected in fringe capacitance level [14].

In general, two-dimensional (2D) materials are recognized as promising sensors with advantages such as high surface area and surface functionality. Ultra-thin materials such as palladium nanosheets (PdNS) and MXene (2D transition metal carbides, nitrides, and carbon nitride family) are trending nanocomposite materials for many types of gas and humidity sensing [15,16,17]. However, there are limitations to the mass production of nanomaterials with high surface area as well as uniform quality. In recent years, MXenes have attracted extensive attention in the field of sensors for their hydrophilic surface, large specific surface area, and high metal conductivity [14]. However, the interaction between layers is a covalent and ionic bond, making it difficult to prepare them by mechanical stripping. So, MXene is prepared by selectively etching the relatively weak metal bond M-A, then peeling off the “A” atomic layer through strong acid or other methods [18]. It can be noted that the reason why graphene can be successfully prepared in the early stage is that the layers of graphene are connected by the van der Waals force. As the humidity increases, the distance between MXene layers increases, increasing the tunnel resistance [19]. By reducing the humidity, the distance between layers is reduced and the resistance is restored. Thus, the main mechanism in Mxene is the change of layer spacing [17]. Hence, maintaining the layer-by-layer spacing in Mxene without water intercalation at high humidity levels is a major limitation. Generally, MXene is prone to degradation when it encounters oxygen and water in the air, reducing its long-term stability performance under highly humid environmental conditions [20,21,22]. This requires more maintenance of material after preparation. In addition, although some studies reported that MXenes are not toxic, the main components of MXene are Carbide or Nitride elements [23]. This could limit the sensing of humidity for biomedical sensing applications. Moreover, MXenes prepared by the bottom-up method are relatively low, so it is not easy to prepare MXenes in large quantities [23,24]. Thus, bulk manufacturing of 2D nanomaterials is a continually challenging issue in industries, which predominantly apply polymers as a sensing material for humidity. However, polymers lack long-term stability and inevitable hysteresis. Moreover, polymers are being used in capacitive humidity sensors which require top electrodes. Most polymer-based humidity sensors operate at room temperature since they are very sensitive to heat. These limitations in polymers have been compensated using ceramic sensing layers as their sensing principle is quite similar to that of polymers. It is reported that ceramic sensing materials such as Graphene Oxide (GO) and Zinc Oxide based humidity sensors are of more interest for precision-equipment manufacturing applications as well as health monitoring [25,26]. Protons generated through the reaction of water molecules with the GO surface functional groups cause the electrical impedance of the material to decrease. This property of GO contributes to a wide field of research as a humidity-sensing material. Though GO-based sensors exhibit fast response and recovery times with little hysteresis, they need a high working voltage (5 V), which may result in high power consumption, especially in the case of micro-sensor design [27]. Moreover, pure GO is highly sensitive to other gases including NO_2_, NH_3,_ and so on. This may affect the overall single-molecule sensitivity of the sensing layer. There are metals such as Zn, Cu, Sn, Au, and Pt that can be added to GO to modify the conductivity and consequently the selectivity of gases [28,29]. Few research works claim that water intercalation in GO contributed to wearing layers at a relative humidity as low as 30% [30]. In high humidity regions, undoped GO might delaminate because of swelling layers. The friction of the SiO_2_/GO interface in an interdigitated electrode capacitive/resistive sensor is affected, when relative humidity is increased. Hence, the lifetime stability of sensing material is very much limited. It is observed from the literature that ZnO is one of the preferred materials because it exhibits superior electrical conductivity, high thermal, chemical stability, mechanical stability, and low cost due to its abundance in nature [31]. Few evaluation results show that nanostructured ZnO can enhance water vapor sensitivity because of increased surface area. Many experimental research works report doping metal oxides with various transition metals (Mn, Co, Li, etc.,). However, Mn-doped ZnO nanopowders displayed long-term reproducibility, stability as well as high sensitivity at room temperature when considered for practical high-performance humidity sensors [32]. It has also been reported that Mn doping would cause the deformation of ZnO and lead to higher free electron density on the ZnO surface. With the ionic radii of Zn^2+^ ion being 74 pm and that of Mn^2+^ being 70 pm, the overall size of the Mn_x_Zn_1−x_O samples decreases with increasing Mn doping concentration. Moreover, the doping of ZnO with transition elements leads to weak structural disorder, which is due to mass and size asymmetries of dopants into the host lattice. Hence, nanosynthesis materials still have challenges to improve long-term stability and effective bulk production. The overall efficiency of humidity sensing could be enhanced by synthesizing nanocomposites. The selection of nanocomposites of GO may significantly increase the performance of the sensor, as GO nanosheets contain rich oxygen groups for good adsorption of water molecules. To avoid the swelling of the sensing layer at higher humidity levels, the in-plane capacitive type sensor is preferred over the sandwich-structured capacitive sensor. It is observed that the time taken for complete desorption of the water molecules in the sandwich structure is more because of top electrode placing. Hence, it is more effective to cast the sensing material on the electrode surface as an in-plane capacitive/resistive type humidity sensor. Mn_x_Zn_1−x_O would form cross-linking between layers of GO to prevent water intercalation and swelling which is detailed in the final session. Moreover, the conduction of GO at low relative humidity (RH%) levels shall be improved, with ZnO and Mn doping in the composite for linear response. This further increases the consistent slope of sensitivity of the sensor for a wider sensing range of RH% levels.

In this work, a highly stable and sensitive in-plane capacitive/resistive humidity sensor based on GO-Mn_x_Zn_1−x_O nanocomposite structures, with relatively facile technology, is developed through freestanding chemical synthesis. Reduced GO nanoparticles and Mn_x_Zn_1−x_O nanoparticles were also synthesized for comparison of performances. All three materials (GO-Mn_x_Zn_1−x_O nanocomposite, GO, and Mn_x_Zn_1−x_O nanoparticles) were coated on five customized different configurations of interdigitated comb electrodes (IDE), with a fixed 6 mm × 3 mm area (but a varied number of fingers, width, and spacing) to choose optimized performance characteristics. The GO-Mn_x_Zn_1−x_O nanocomposite sensing layer is also tested for different humidity levels and characterized using various characterization techniques. The enhanced performance of the sensing device is predicated due to the peculiar characteristic of GO-Mn_x_Zn_1−x_O nanocomposites.

## 2. Materials and Methods

Initially, an extensive analysis is carried out to synthesize nanocomposite-containing GO as an essential compound. Hence, the whole synthesis process is performed at relatively low-temperature levels (less than 600 °C) compared to ceramic metal oxides, which usually can withstand temperatures above 800 °C [3].

### 2.1. Material Synthesis

Graphene Oxide (GO) is prepared by oxidizing graphite powder using the well-established modified Hummer’s method.

#### 2.1.1. Preparation of Mn_x_Zn_1−x_O Nanopowder

Zinc acetate (Zn(CH_3_CO_2_)_2_·2H₂O) and Manganese acetate ((CH_3_CO_2_)_2_Mn) were taken as precursors in the sol-gel process in a 1:1 mole ratio. Each precursor is dissolved in 50 mL of deionized water. These solutions are further dissolved in 30 mL of dimethylformamide at room temperature and mixed to a clear solution under vigorous stirring. The clear solution is further stirred for 24 h, maintaining the temperature between 60 °C to 70 °C. The gel formation is observed after vaporizing. The gel product is then dehydrated at 150 °C for 3 h. Final nanopowder Mn_x_Zn_1−x_O is obtained after thermal decomposition of the dehydrated gel at a calcination temperature of 350 °C for 4 h. The concentration of Mn in the final product is 37%.

#### 2.1.2. Preparation of GO-Mn_x_Zn_1−x_O Nanocomposite

Here, 2.2 g of Zinc acetate and 0.272 g of Manganese acetate (the molar ratio of Mn to Zn is 0.1:0.9) were dissolved in GO (already prepared by Hummers’ method) by adding anhydrous ethanol solution (0.6 g/L). The mixture is stirred and sonicated for about 30 min, after which the solution contains oxalic acid to metal ion molar ratio of 1:1. This solution with 55 mL of anhydrous ethanol is slowly dropped into the graphene oxide solution at 50 °C, producing a precursor. Finally, the precursor is annealed in argon gas starting from room temperature to 600 °C at the rate of 5 °C/min and held for 2 h. The ground finished end product is a dark grey GO-Mn_x_Zn_1−x_O nanocomposite powder with less than 2% Mn as a dopant.

### 2.2. Device Fabrication

**Figure 1:**
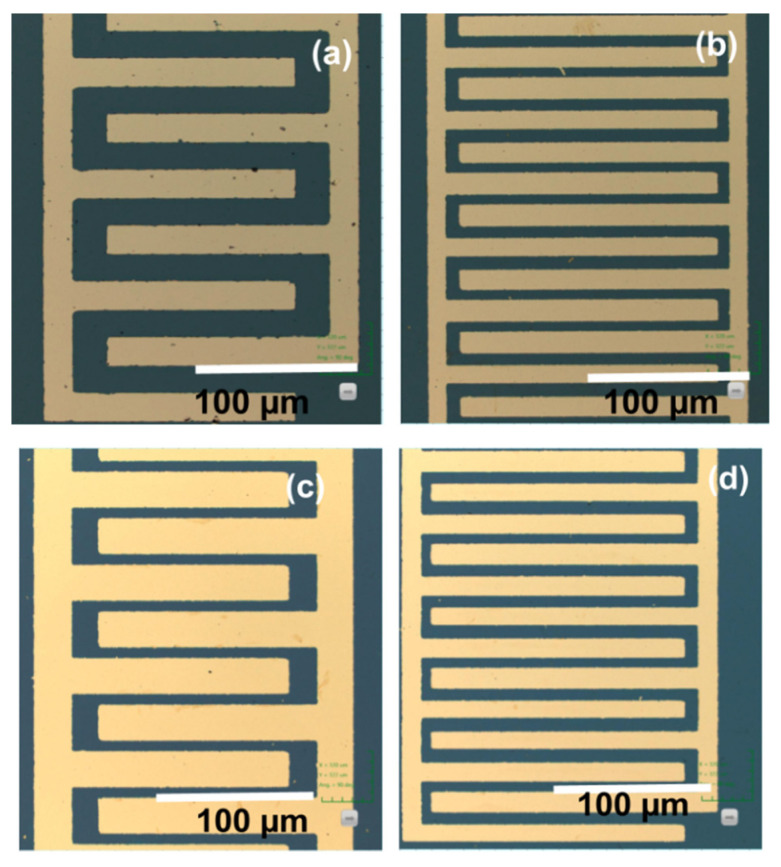
Confocal images of (**a**) 10 fingers and (**b**) 20 fingers by the lift-off method, (**c**) 16 fingers and (**d**) 28 fingers by metal-etching method.

Fabrication of IDE with the variation of finger width and distance between fingers is done by a conventional photolithography technique. A silicon (100) substrate of 4-inch diameter is used on which a 1 µm-thick SiO_2_ layer is grown carefully using thermal oxidation technique at 1000 °C for 4 h including different stages of dry and wet oxidation. The wafer is cleaned with trichloroethylene and isopropyl alcohol followed by acid cleaning with nitric acid and hydrofluoric acid to remove the oxide layer formed in atmospheric conditions. Then, gold (100 nm thick) is deposited using thermal evaporation and its thickness is measured to be an average of 98 nm with roughness less than 2 nm. An effective photomask pattern is designed with different numbers of fingers, finger widths, and different spatial wavelengths (λ), as listed in Table 1. A reversal photomask is also prepared to compare the lift-off process and metal etching. The fabricated sensor is diced into 6 mm × 3 mm. More than 45 fabricated sensors with varying numbers of fingers are taken for testing. A 5 µL sample solution of reduced GO, Mn_x_Zn_1−x_O, and GO-Mn_x_Zn_1−x_O nanocomposite is individually mixed with deionized water and drop cast on fabricated sensors. The required electrical connections are taken out by using silver conductive paste and the sensor devices are mounted on copper-toned FR-4 board. Figure 1a,b details the Confocal images of lift-off electrodes with improper finishing. The metal etching shows better electrode finishing than the lift-off method, as seen in Figure 1c,d. Hence, electrodes processed with metal etching are further taken for coating with sensing layers and testing.

### 2.3. Humidity Sensing Test Set-Up

**Figure 2:**
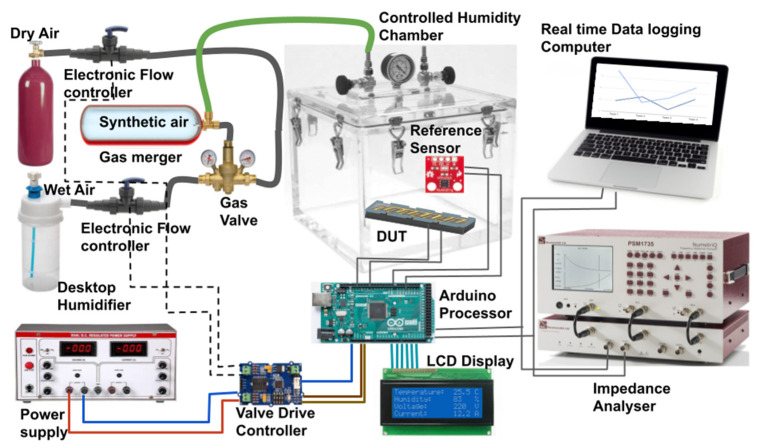
Humidity sensing setup.

The fabricated humidity sensor is characterized by setting up an in-house controlled environment of a 1-L volume chamber maintained at a constant temperature of 25 °C, with the reliable data recording. The schematic diagram of the test set-up is presented in Figure 2. The percentage of relative humidity in the sealed chamber is controlled by an automatic feedback controller. Atomized water (wet air), from a 2 L desktop humidifier, is mixed with dry air, before sending it to the controlled humidity chamber in a gas merger as synthetic air. The humidity level is first set manually to a point till the impedance reading of the sensor is stable. HTU21D humidity sensor (Measurement Specialties Inc, MEAS, France) with a resolution of 0.04%RH is used as a reference sensor and as feedback to the digital controller for sensing current humidity in the chamber [27]. The temperature coefficient for the reference sensor is −0.15%RH/°C and the response time is around 5 s. The valve’s position is determined by the feedback flow controller output. The real-time readings of impedance are sent through USB to the computer for data logging and the same is measured with PSM1735 impedance analyzer (Newtons4th Ltd., Mountsorrel Loughborough Leics, UK) for a wide range of frequencies. Arduino MEGA 2560 (Italy) is used as the main processing board for interfacing with sensors and computers. The AC impedance of all sensors is measured at 100 kHz test frequency and the input signal with 1 V magnitude.

### 2.4. Equipment Incorporated

The following tools are used to characterize the nanomaterials: (i) Scanning Electron Microscope with EDS MA15/ EVO 18 (Carl Zeiss Microscopy GmbH, Jena, Germany) is used to investigate the surface morphology of the nanostructure; (ii) PANalytical X’Pert Powder X-Ray Diffractometer System (Malvern Panalytical Ltd., Malvern, UK) is used to obtain XRD patterns; (iii) Scanning Electron Microscope NanoAnalysis INCA Energy 250 Microanalysis System -EDS (Oxford Instruments, Cedex, Saclay, France) is used for quantitative analysis of synthesized nanomaterials; (iv) Digital Instruments Dimension^TM^ 3100 Atomic Force Microscope(EXW Charlotte, NC, USA) is used for high-resolution height profiling of the comb electrode; (v) Agilent BA1500 parameter analyzer(Agilent Technologies, Inc. Santa Clara, CA, USA) is used for current measurement.

The sensor fabrication process is done with the following equipment: (i) oxidation of p-type(100) silicon wafer was done in the Tempress Omega three heating zone chamber(Omega Engineering, Inc., Norwalk, CT, USA); (ii) Mask Writer Heidelberg—DWL 66 (Heidelberg Instruments Mikrotechnik GmbH, Heidelberg, Germany) is used to prepare positive and negative masks for the lift-off and metal etching processes, respectively; (iii) Photolithography Mask Aligner Model 5000 (OAI, Milpitas, CA, USA) was used for lithographing; (iv) E-beam Evaporation BOC Edwards Auto 306 (BOC Edwards, Crawley, UK) is used to pattern gold for electrodes; (v) 7100-Provectvs (Advanced Dicing Technologies Inc., Horsham, PA, USA) is used for precise dicing of multiple sensors of 6 mm × 3 mm each from a 4-inch × 4-inch wafer; (vi) detailed patterns of different dimensions of comb electrodes are studied using Confocal Microscope Olympus LEXT 3D Measuring Laser Microscope OLS4000 (Olympus Corporation, Tokyo, Japan); (vii) thickness of oxide layer and gold electrode layers are measured using Woollam Spectroscopic Ellipsometer M-2000VI EC-400 (J.A. Woollam Co., Inc., Lincoln, NE, USA) focusing beam angle kept at <70°.

## 3. Results and Discussion

### 3.1. Material Characterization

**Figure 3:**
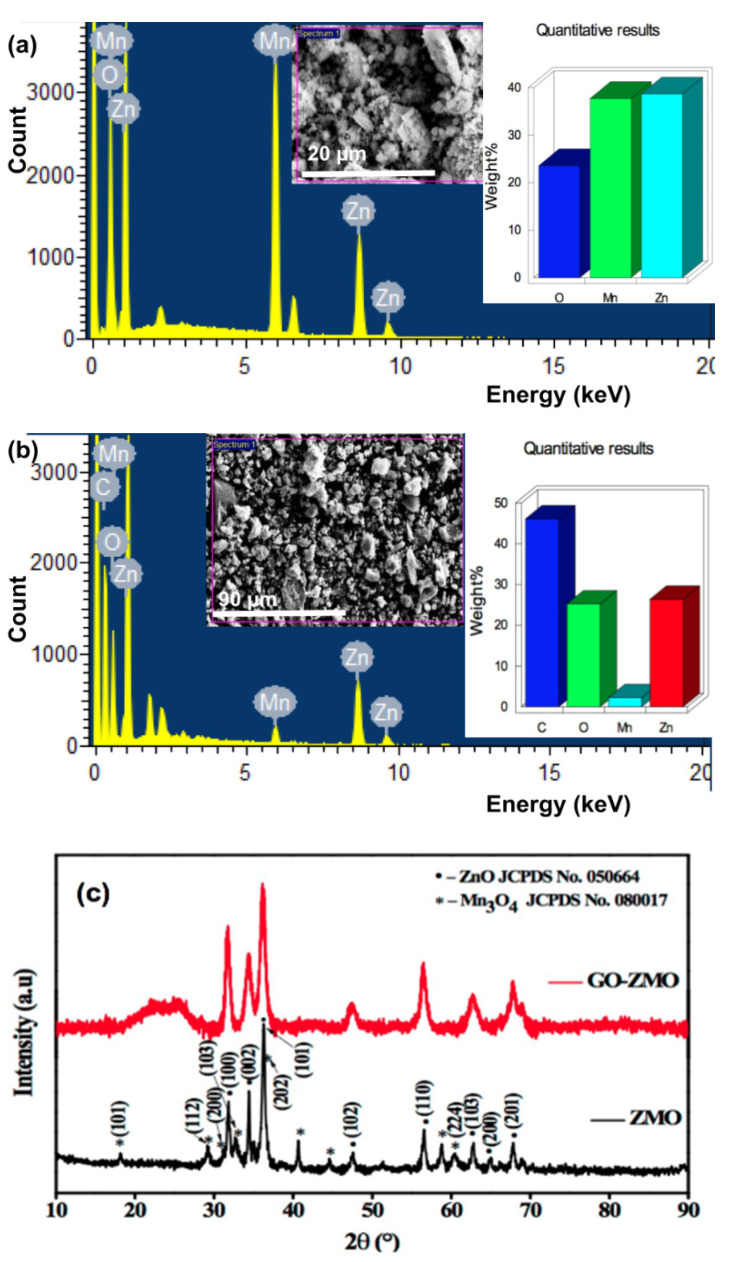
(**a**) EDS XRD of Mn_x_Zn₁_−x_O with (inset) showing the image of Mn_x_Zn_1−x_O and quantitative analysis revealing 37.7% of Mn (**b**) EDS XRD of GO-Mn_x_Zn_1−x_O nanocomposite (inset) shows SEM image along with quantitative analysis of GO-Mn_x_Zn_1−x_O nanocomposite; the Mn and Zn constituted 2.23% and 26.39%, respectively, (**c**) HR-XRD of Mn_x_Zn_1−x_O and GO-Mn_x_Zn_1−x_O nanocomposite.

The Energy Dispersive X-ray Spectrometry (EDS) data reveal the concentration of Mn_x_Zn_1−x_O and GO-Mn_x_Zn_1−x_O powders, as shown in Figure 3a,b with insets. It is found that the composition of Mn_x_Zn_1−x_O has 37.7% of Mn. In GO-Mn_x_Zn_1−x_O nanocomposite, the Mn and Zn constituted 2.23% and 26.39% respectively. In Figure 3b, the Carbon(C) signal peak originates from GO. The Mn and Zn signals authenticate the presence of the nanoparticles in the GO sheet. In this composition, the GO constitutes a major value of 71.37%, which is revealed by major peaks in Figure 3b. The XRD study is performed with a CuKɑ source and the wavelength used is 1.54060 Å. Figure 3c displays the HR-XRD of Mn_x_Zn_1−x_O and GO-Mn_x_Zn_1−x_O peaks. The broadening of the peak near 2θ = 24.5° shows a reduction of GO, hence forming a composite. The peaks are sharper at (100), (002), (101), (102), (110), and (201) planes. It is found that the samples exhibiting the diffraction peaks of ZnO and Mn_3_O_4_ are in good agreement with JCPDS no.050664 and JCPDS no.080017.

**Figure 4:**
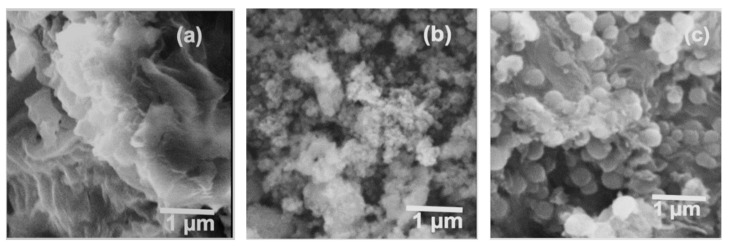
SEM images of (**a**) GO (**b**) Mn_x_Zn_1−x_O and (**c**) GO-Mn_x_Zn_1−x_O.

To analyze the morphological structure of GO, Mn_x_Zn_1−x_O, and GO-Mn_x_Zn_1−x_O nanocomposite, SEM images are studied. The sheet structure of reduced GO is seen in Figure 4a. The image of Mn_x_Zn_1−x_O in Figure 4b reveals that the particle sizes are ranging from 55 nm to 80 nm.

The SEM image of nanocomposite GO-Mn_x_Zn_1−x_O in Figure 4c shows the Mn_x_Zn_1−x_O in GO sheets, with particle sizes ranging between 120 nm–140 nm. Mn_x_Zn_1−x_O nanoparticles are tightly adsorbed on GO sheets.

**Figure 5:**
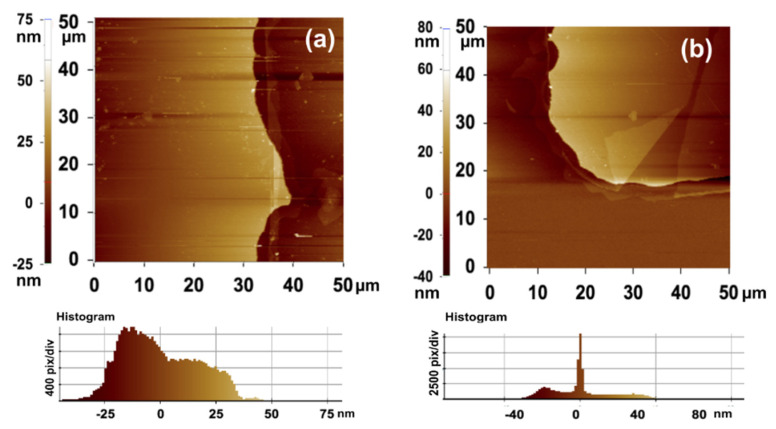
AFM height profile of (**a**) comb-step thickness and (**b**) comb-edge height profile.

Since the sensing layer is in-plane with the interdigital electrode, the fringing electric field penetration in the sensing material plays an important role in measuring capacitance change with relative humidity, which is proportional to comb electrode dimensions [33,34]. Hence, the etching finish of the comb finger is also considered for the overall performance of the sensor [35]. Therefore, to analyze the surface portfolio of comb structure fabrication, an AFM image of the metal-etched sensor is taken and shown in Figure 5. In Figure 5a, the comb step thickness is found to be 35 µm, and Figure 5b reveals the comb edge height of 40 µm. These results show the effective deposition and metal etching of the gold comb, which may be used for bulk production and also gives an even distribution of electric field over the sensing layer.

### 3.2. Humidity Sensing

In this work, a capacitive/resistive humidity sensing device is designed by drop-casting the sensing material on a custom-designed IDE. Though the comb electrodes are designed in five different combinations of numbers of fingers, the spatial wavelength of less than 0.6 mm gives better performance; hence 28, 20, and 16 finger combs are taken for further detailed testing. This is because the spatial wavelength is proportional to the penetration depth of fringing electric fields above IDE [36]. The detailed analysis is discussed in the final session of this paper.

**Figure 6:**
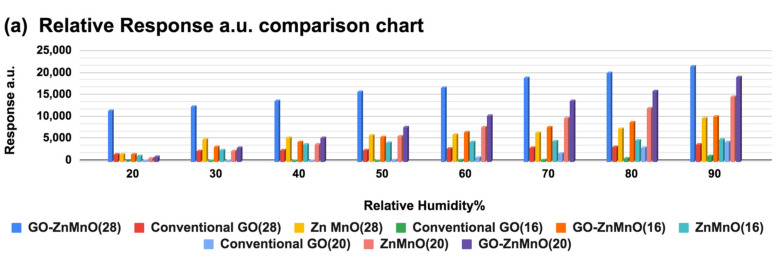

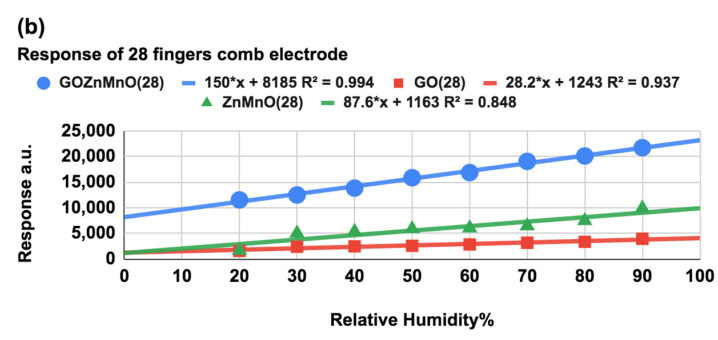
(**a**) Response comparison chart for GO, Mn_x_Zn_1−x_O, and GO-Mn_x_Zn_1−x_O on 28-, 20-, and 16-finger combs. (**b**) Specific 28-finger comb response with a linear fit.

To obtain a stable baseline, the device is exposed to 15% RH compressed air for half an hour. For a change in RH%, the output current changes relatively. This change is considered response of the device [37]. The response from the device is calculated using Equation (1):

(1) $$\;\;Response=\frac{I_{RH}-I_{base}}{I_{base}}$$

where *I_base_* corresponds to current at 15%RH, while *I_RH_* is the current at respective measured RH%. It is observed that the response for 28 fingers GO-Mn_x_Zn_1−x_O (mentioned as GOZnMnO in all graphs) nanocomposite is much higher than GO and Mn_x_Zn_1−x_O (mentioned as ZnMnO in all further graphs). The overall response comparison with corresponding RH% change is shown in Figure 6a. The response is measured for different RH% (20% to 90% at a difference of 10%RH), which proves a great achievement of a wide sensing range of humidity. At 40%RH, GO-Mn_x_Zn_1−x_O nanocomposite enhanced the response 82.67 times more than GO, and 61.28 times more than Mn_x_Zn_1−x_O (plotted in Figure 6b with a linear fit). This enhancement of response is consistent till the 90%RH range.

**Figure 7:**
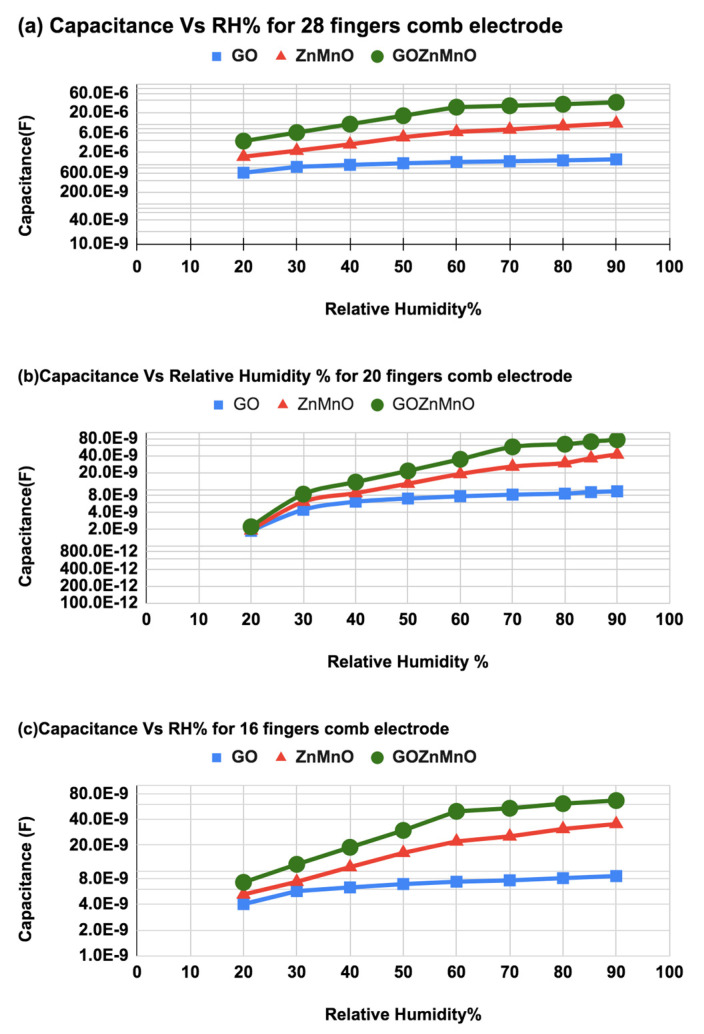
GO-Mn_x_Zn_1−x_O, Mn_x_Zn_1−x_O and GO for (**a**) capacitance vs. RH% for 28-finger comb electrode, (**b**) capacitance vs. RH% for 20-finger comb electrode, (**c**) capacitance vs. RH% for 16-finger comb electrode.

**Figure 8:**
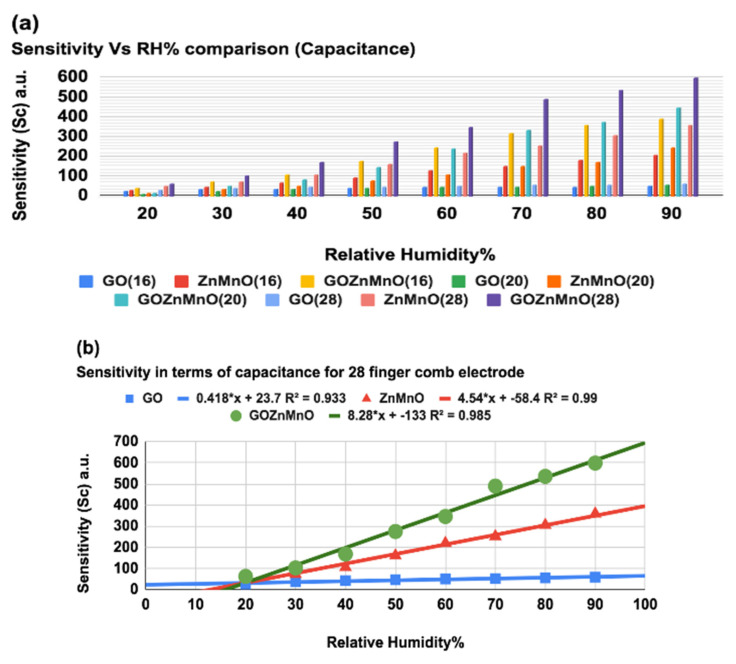
(**a**) Sensitivity vs. RH% comparison chart for GO-Mn_x_Zn_1−x_O nanocomposite, Mn_x_Zn_1−x_O and GO for 28-, 20-, and 16-finger comb electrodes in terms of capacitance. (**b**) Sensitivity ($S_{C}$) as a function of RH% for 28 fingers comb electrodes with a linear fit.

Sensitivity is a critical parameter in choosing any sensor. This fabricated sensor sensitivity is tested for two parameters, namely capacitance and resistance, relative to the change in RH%. Initially, sensitivity is studied in terms of capacitance for various ranges of RH%. The variation of capacitance for 28, 20, and 16 finger combs at 100 kHz frequency and 1 V AC input signal are taken separately for GO-Mn_x_Zn_1−x_O nanocomposite, Mn_x_Zn_1−x_O, and GO, as shown in Figure 7. Figure 7a shows the enhanced value of capacitance for 28-finger IDE with GO-Mn_x_Zn_1−x_O nanocomposite, which is 72.2 times more than Mn_x_Zn_1−x_O and 95.7 times higher than conventional GO at 60%RH, respectively. Similarly, from Figure 7b,c, it is observed that the GO-Mn_x_Zn_1−x_O nanocomposite sensing layer has maximum capacitance for a wide range of RH%. The dependence of capacitive sensitivity of the sensor on the sensing layer to the number of fingers in comb electrodes concerning a wide range of RH% change is studied. Figure 8a displays the comparison chart for sensitivity with RH%. The reported data are mean values taken from several measurement cycles maintaining a temperature of 25 °C, for a 1 V AC signal with 100 kHz frequency. As per the IUPAC definition, the sensitivity ($S_{C}$) can be defined as the relative capacitance difference over the relative humidity difference. To depict sensor sensitivity in terms of capacitance [27], the formula is defined as in Equation (2):

(2) $$\;Sensitivity\left(S_{C}\right)=\frac{\left(\frac{C_{x}-C_{15}}{C_{15}}\right)}{\%RH_{x}-\%RH_{15}}$$

where *C_x_* and *C*_15_ are capacitances at current RH% and 15% RH levels respectively and *%RH_x_* is current RH% under investigation. It is depicted in Figure 8a that GO-Mn_x_Zn_1−x_O nanocomposite has the highest sensitivity for 28-finger comb electrode. The sensitivity ($S_{C}$) is compared with Figure 8b, which reveals that the newly developed nanocomposite has 85 times greater sensitivity than GO and 36 times more sensitivity than Mn_x_Zn_1−x_O at 60%RH for the 28-finger comb electrode. It is evident from Figure 8b that the sensitivity varies consistently (fitted with y = a + bx; a= −133; b = 8.28; Adj.R^2^ = 0.985).

**Figure 9:**
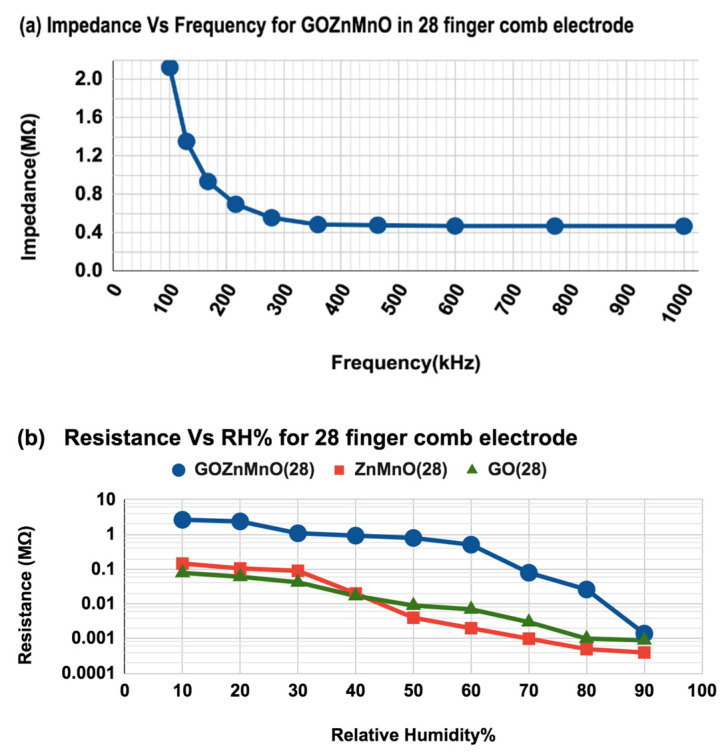

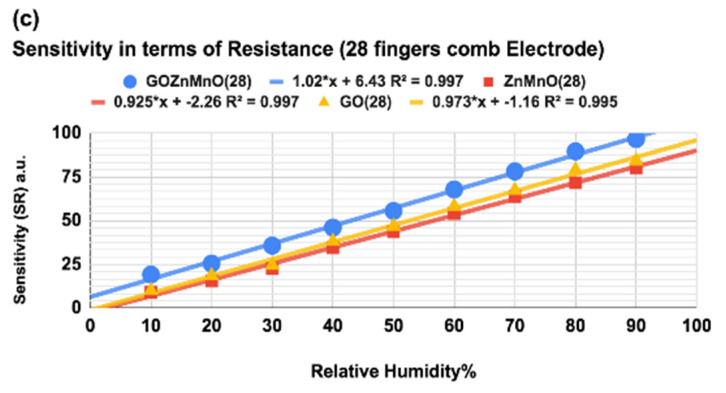
(**a**) Change in impedance with frequency at 50%RH and room temperature of 25 °C for GO-Mn_x_Zn_1−x_O nanocomposite, (**b**) sensitivity vs. RH% comparison chart for GO-Mn_x_Zn_1−x_O nanocomposite, Mn_x_Zn_1−x_O and GO for 28-finger comb electrodes in terms of resistance (Ω). (**c**) Defined sensitivity (*S_R_*) as a function of RH% for 28-finger comb electrode in resistance (Ω) with a linear fit.

As depicted in Figure 9a, the impedance decreases with increasing frequency. The change in impedance is more pronounced at 100 kHz. Beyond 500 kHz, the variation in impedance is much smaller because the polarization of water molecules is limited at higher frequencies [12]. Hence, 100 kHz frequency is chosen as the test frequency for the sensor [13]. The change in resistance of the sensing material is also taken for the analysis of the sensor characteristics as represented in Figure 9b. It is observed that the resistance range changes on a logarithmic scale for an increase of RH%, making the sensor reliable as a resistive type of humidity sensing. GO-Mn_x_Zn_1−x_O shows a wide change in resistance, ranging from 2.6 MΩ to 1.4 kΩ at 10%RH to 90%RH, respectively. This proves the enhanced performance of the nanocomposite is 94.5 times higher than Mn_x_Zn_1−x_O and 97 times greater than the conventional GO.

As per the IUPAC definition, the *sensitivity* ($S_{R}$) can be expressed as the relative resistance difference over the relative humidity difference. The sensitivity of material in terms of change in resistance is formulated using Equation (3):

(3) $$Sensitivity\left(S_{R}\right)=\frac{\left(\frac{\left(R_{0}-R_{h}\right)}{R_{h}}\right)}{\%RH_{x}-\%RH_{10}}\times 100\%$$

where *R*_0_ is resistance at 10% RH and *R_h_* is resistance at current RH% under investigation; while *%RH_x_* is current RH% under investigation and *%RH*_10_ is 10% relative humidity [32]. Figure 9c interprets the linear rise of the *sensitivity* (*S_R_*) for wide range of change in RH% (fitted with y = a + bx; a = −6.43; b = 1.02; Adj.R^2^ = 0.997). Moreover, the new nanocomposite depicts a 10.74-times enhancement of sensitivity than Mn_x_Zn_1−x_O and 15.5 times more than that of the GO material.

**Figure 10:**
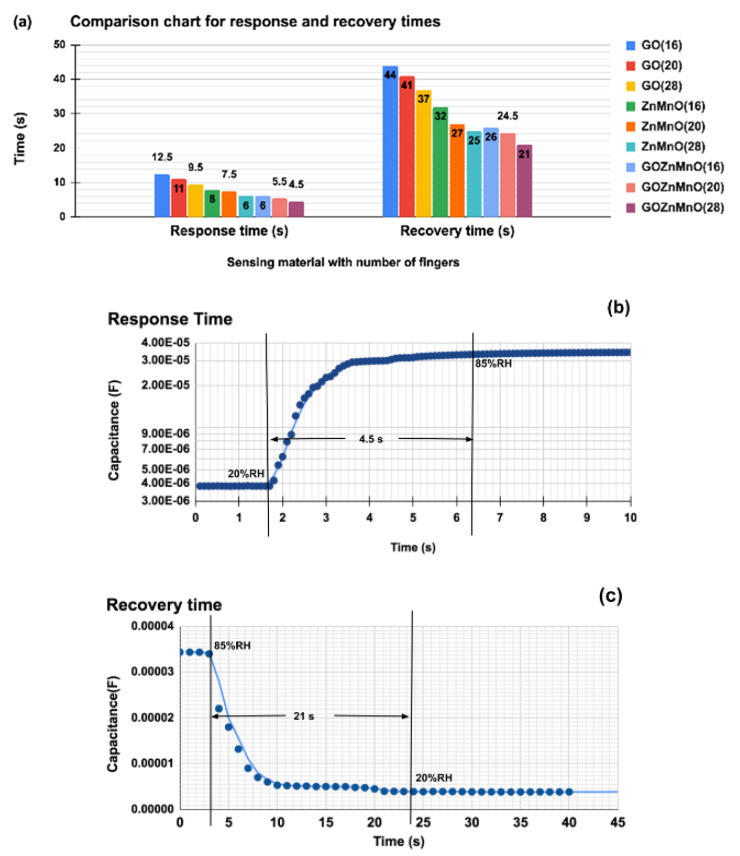
(**a**) Comparison chart for response and recovery time GO-Mn_x_Zn_1−x_O nanocomposite, Mn_x_Zn_1−x_O, and GO for 16-, 20-, and 28-finger comb electrodes in terms of capacitance. (**b**) Response time for humidification from 20%RH to 85%RH (~4.5 s). (**c**) Recovery time for desiccation from 85%RH to 20%RH(~21 s) for GO-Mn_x_Zn_1−x_O nanohybrid with 28-finger comb electrode.

In this research work, the comb electrode dimensional parameters and sensing layer properties significantly affect response and recovery times. Both response and recovery times are defined with RH% ranging from 20% to 85% while increasing as well as decreasing RH%. A detailed comparison of response times and recovery times for GO-Mn_x_Zn_1−x_O nanocomposite, Mn_x_Zn_1−x_O, and GO for 16-, 20-, and 28-finger comb electrodes in terms of capacitance is shown in Figure 10a. The length, spacing, and width of the comb electrodes (IDE) are the same, with the number of fingers changing between 16, 20, and 28.

The spacing of the electrodes is inversely proportional to the capacitance, while the sensing material dielectric properties and the number of fingers in the comb electrode are directly proportional to the capacitance of the humidity sensor [28]. This shows the overall optimized number of fingers as 28, and the sensing material GO-Mn_x_Zn_1−x_O nanocomposite performed better for further analysis of the response and recovery times.

As per recorder plots, demonstrated in Figure 10b,c, the humidification shows response time as ~4.5 s, and desiccation shows recovery time as ~21 s for GO-Mn_x_Zn_1−x_O nanocomposite with 28 fingers. This is a reliable improvement, compared with conventional GO (response time ~10.5 s and recovery time ~41 s) and Mn_x_Zn_1−x_O (response time ~6 s and recovery time of ~25 s) [31,32]. Hence, the newly prepared nanocomposite shows a comparable and faster response time for humidity sensing. It has to be noted that humidity in any given environment cannot change drastically in milliseconds. However, individual nanomaterials such as GO, Mn_x_Zn_1−x_O are used to detect other gases such as NO_2_, CO_2,_ etc., but the responses for gas detection are comparatively lower than humidity sensing [3]. This is because of the physisorption and chemisorption of water molecules in the GO layer in the GO-Mn_x_Zn_1−x_O nanocomposite.

**Figure 11:**
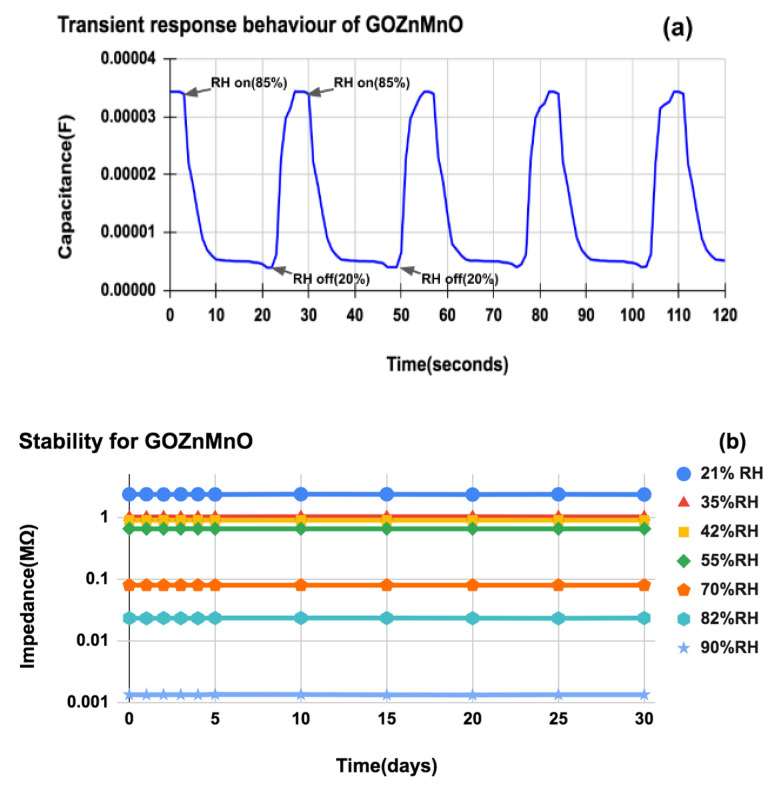
(**a**) Transient response behavior of GO-Mn_x_Zn_1−x_O nanocomposite-based humidity sensor with 28-finger comb electrode to dynamic switches between 20%RH and 85%RH (**b**). Stability of GO-Mn_x_Zn_1−x_O (after 5 months of aging) in terms of impedance (MΩ) change to time (days) for various RH% levels.

The transient response of the GO-Mn_x_Zn_1−x_O nanocomposite-based humidity sensor is illustrated in Figure 11a. The dynamic switching between 20%RH and 85%RH is performed. The humidity sensor when exposed to air of 85%RH about 20%RH shows a prompt decrease of capacitance and reaches a relatively stable value and increases instantly when switched from 20% to 85%RH. An additional five cycles are carried out and the results show good reproducibility.

The stability of the sensor is one of the most important parameters in analyzing humidity sensing for a long period. When nanomaterials play the sensing role, their agglomeration is removed by simple mortar and pestle grinding. Considering the stability, the GO-Mn_x_Zn_1−x_O nanocomposite is aged for more than 5 months and tested repeatedly under different humidity levels (21%, 35%, 42%, 55%, 70%, 82%, and 90%) for 30 consecutive days. The impedance is recorded at 100 kHz frequency and shows variations of less than 4% as seen in Figure 11b. This data shows very good consistency for a long period of testing, which highlights that newly synthesized nanocomposite is liable for bulk industrial productions. The samples are again dried to observe any peel-off of the sensing layer due to water intercalation. It is found to be in good condition as tested initially, making the layer bonding between the silicon substrate and the in-plane sensing layer stronger.

**Figure 12:**
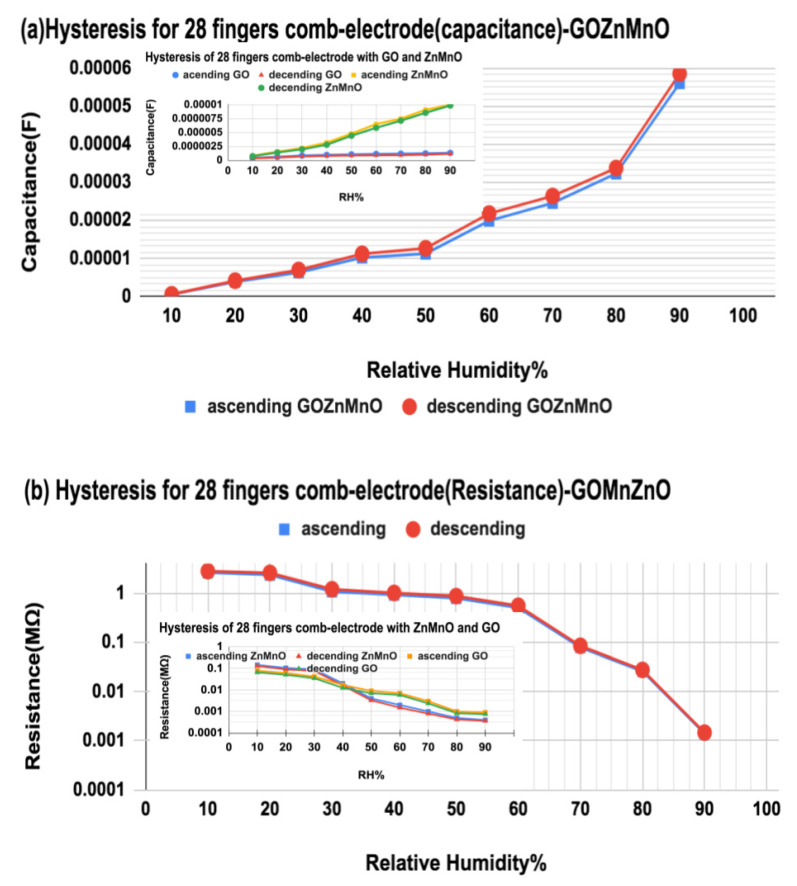
(**a**) Hysteresis error in terms of capacitance for humidification ascending and descending from 10%RH to 90%RH); inset shows the hysteresis error of Mn_x_Zn_1−x_O and GO. (**b**) Hysteresis error in terms of impedance descending and ascending from 90%RH to 10%RH for GO-Mn_x_Zn_1−x_O nanohybrid with 28-finger comb electrode; inset shows hysteresis error of Mn_x_Zn_1−x_O and GO with 28-finger comb electrode.

The hysteresis error plays a very important role in the humidity-sensing property of the sensor as it defines the adaptability of humidity sensors for various environments. Since GO is hydrophilic, the synthesized GO nanocomposite shows less hysteresis error, as given in Figure 12. Both capacitance change and impedance change are recorded for ascending and descending RH% from 10% to 90%. The maximum value of capacitance error is 0.125 at 40%RH, and a minimum of 0.045 at 90%RH in capacitance, for GO-Mn_x_Zn_1−x_O nanocomposite as demonstrated in Figure 12a. The inset in Figure 12a shows the hysteresis error of GO with a maximum error of 0.2 at 60%RH and a minimum of 0.11 at 90%RH while Mn_x_Zn_1−x_O has a hysteresis error of maximum of 0.12 at 40%RH and minimum of 0.06 at 90%RH. Similarly, the maximum error in impedance for GO-Mn_x_Zn_1−x_O nanocomposite is 0.105 at 50%RH and a minimum of 0.062 at 90%RH in impedance as shown in Figure 12b. The inset in Figure 12b shows the hysteresis error of GO as 0.27 maximum at 60%RH and minimum of 0.17 at 90%RH while Mn_x_Zn_1−x_O shows a maximum hysteresis error of 0.25 at 40%RH and minimum of 0.09 at 90%RH. 

These experimental results demonstrate the excellent performance of an in-plane capacitive/resistive humidity sensor, coated with an effectively synthesized GO-Mn_x_Zn_1−x_O nanocomposite sensing layer. The probable reasons behind the performance analysis in sensing the humidity sensor are the doping of nanocomposite, and the effects of choosing the dimensions of comb electrode (IDE) for optimizing the measuring parameters, which are thereby discussed analytically.

## 4. Discussion

### 4.1. The Probable Humidity Sensing Mechanism

**Figure 13:**
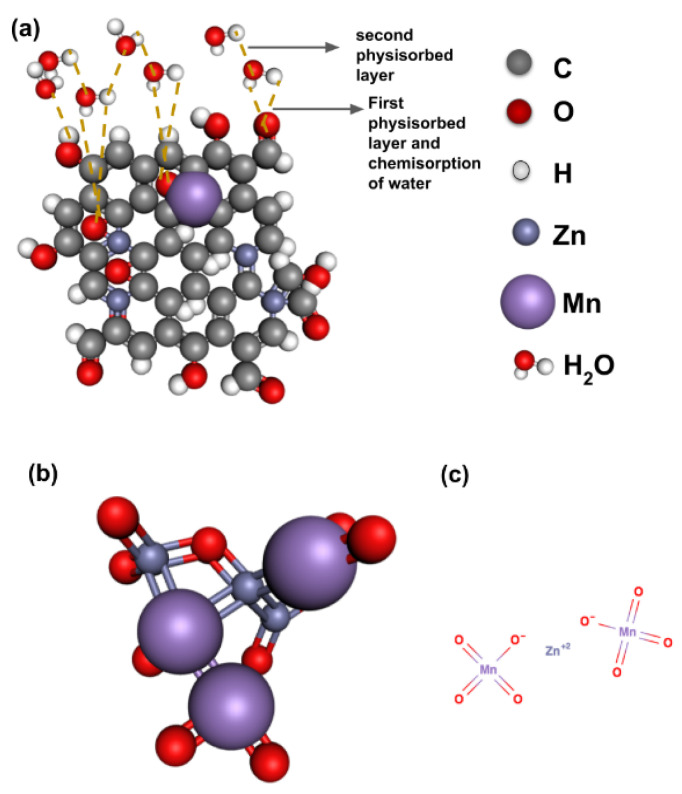
(**a**) Structure of GO deformation with Zn and Mn dopants in the nanocomposite GO-Zn_1−x_Mn_x_O with oxygen-rich groups and tailored defects enhancing water absorption. (**b**) Nucleation of Mn particles in the ZnMnO compound. (**c**) Dislodging of Zn by Mn atoms to increase states near the conduction band.

GO produced through Hummer’s method accounts for a hydrophilic character with a high density of oxygen functional groups. GO opens the bandgap of graphene which is done through versatile defect engineering. On reducing the GO, some oxygen groups are removed and thus the energy band gap is adjusted by managing the oxygen groups present [38,39]. The bandgap of pure ZnO is 3.3 eV. To enhance single-molecule sensitivity and electrical properties, ZnO is modified with transition metals. ‘Mn’ being 3d metal, is most preferred here since it increases surface area and reduces the particle size of ZnO. The electron effective mass of Mn is approximately 0.3m_e_ (m_e_ is free electron mass), when doped causes a large carrier and injected spins. Figure 13a shows the modified structure of our GO-Mn_x_Zn_1−x_O nanocomposite.

In the case of synthesized Zn_1−x_Mn_x_O nanoparticles, a higher concentration of Mn(37 w%) increased the agglomeration, because of the kinetic equilibrium process. The high concentration of Mn dopants might be nucleated with oxygen ions by itself and formed bigger particles as illustrated in Figure 13b. This could be the reason for the incorporation difficulty of Mn in the ZnO lattice in the Zn_1−x_Mn_x_O compound [40].

GO is widely known for high electrical resistance, especially at lower values of RH%. During the first layer of physisorption of water molecules and double hydrogen bonding, it requires high energy for conduction through proton hopping transfer below 20%RH as visualized in Figure 13a. However, there is leak conduction, which increases capacitance even at low RH% [41,42,43]. Moreover, water intercalation in bulk GO layers is almost double the thickness at 80%RH [44]. To overcome these limitations, Mn is doped with a Zn lattice structure which is tailored to the GO structure.

The concentration of Mn in the newly synthesized nanocomposite is taken to less than 2% mol to tune the energy bandgap in the GO-Mn_x_Zn_1−x_O nanocomposite. The synthesizing is done at low temperature and the process is a wet chemical method which, on the formation of MnO_2_, would eventually lower the effective bandgap [45,46]. Mn dislodges the Zn atom to create energy states close to the conduction as shown in Figure 13c. This may be the reason for the better conductivity of the nanocomposite at low levels of RH% (the energy gap of GO is approximately 2.2 eV).

With the comparison of reduced GO nanoparticles, Zn_1−x_Mn_x_O compound, and the newly synthesized GO-Mn_x_Zn_1−x_O nanocomposite, the added dopants crosslinked GO with silicon substrate even at high RH%(>85%), thus improving the stability. It further enhanced the electrical conductivity at low values of RH%(<20%). The ease of measurement for a wide range of RH% increased, and the longevity of the sensing layer for continuous monitoring. In addition, it reduces recovery time to nearly half (21 s) compared to pristine GO (41 s) [47].

### 4.2. Effect of Comb Electrode Parameters on Sensitivity

The major advantages of interdigital electrodes are fast measurement, being non-destructive, non-intrusive, and possessing compatibility for continuous measurements [48,49]. At constant temperature, relative humidity, and frequency, it is observed that the dielectric constant, impedance, as well as sensitivity, varies for the sensor with change in comb width, comb spacing, and the number of comb fingers [50].

The reduction of interface impedance between the sensing layer and electrodes improves the applied frequency bandwidth for measurement [44,51]. Hence, the cell factor (*K_cell_*) is taken into consideration for comparing the electrode dimensions that are given by Equation (4) to Equation (10):

(4) $$K_{\mathrm{cell}}=\frac{2}{L\left(N-1\right)}\cdot \frac{K\left(k\right)}{K(\sqrt{1-k^{2})}}$$

where *L* is the length of an electrode (mm), *N* is the number of the electrodes of the sensor, W is the width of an electrode (μm), *K_cell_* is the factor of the cell (m^−1^) [31].

*K(k)* is then given by,

(5) $$K(k)=\int_{0}^{1}\frac{1}{\sqrt{\left(1-t^{2}\right)\left(1-t^{2}k^{2}\right)}}\mathrm{dt}$$

(6) $$\mathrm{where}\;k=\cos(\frac{\pi }{2}\cdot \alpha )$$

α is metallization ratio

The resistivity (*R_s_*) of the sensing layer given by Equation (7) is inversely proportional to the cell factor *K_cell_*.

(7) $$Rs_{\;}=\frac{K_{cell}}{\sigma s}$$

where *σs* is the electric conductivity of the sensing medium (S/m).

The total capacitance (C_t_), at the interface of the surface of sensor electrodes, and sensing layer is given by Equation (8),

(8) $$Ct_{\;}=\frac{N}{4}{\mathrm{LWC}}_{0}$$

where C_0_ is the capacitance per unit area (pF/μm^2^) given by Equation (9),

(9) $$C_{0}=\;\frac{4\varepsilon 0\varepsilon r}{NLWK_{cell}}$$

The impedance of polarization which appears at the contact surface between electrodes and sensing layer is determined by the following Equation (10):

(10) $$\mathrm{Zp}\;=\frac{1}{j\omega \mathrm{Ct}}$$

Hence, to choose an effective comb electrode, the effect of various dimensions are studied and tabulated in Table 2. Moreover, from Equation (9), it is observed that the width (W), number of fingers (N), and, thereby, in-plane area of the electrode play a major role in interface capacitance. This could be the reason for obtaining higher values of interface capacitance C_t_ (1932 pF and 1995 pF) for 10 and 14 fingers (W = 0.2 mm), while compared to 16 fingers with width W = 0.1 mm, having a capacitance of 1607 pF. Moreover, optimizing the in-plane area, with the number of fingers (N) as 28, shows enhanced overall comb dimensions for effective bulk production methods (such as lift-off and metal-etching) of sensor electrodes. According to Equation (10), the interface capacitance C_t_ is inversely proportional to polarization impedance (Z_p_). From the above demonstrations, interface capacitance (C_t_) is 2901 pF for 28 finger electrodes, which reduces the polarization impedance more than 1/1000 times, and confirms the enhanced sensitivity at an operating frequency of 100 kHz.

**Figure 14:**
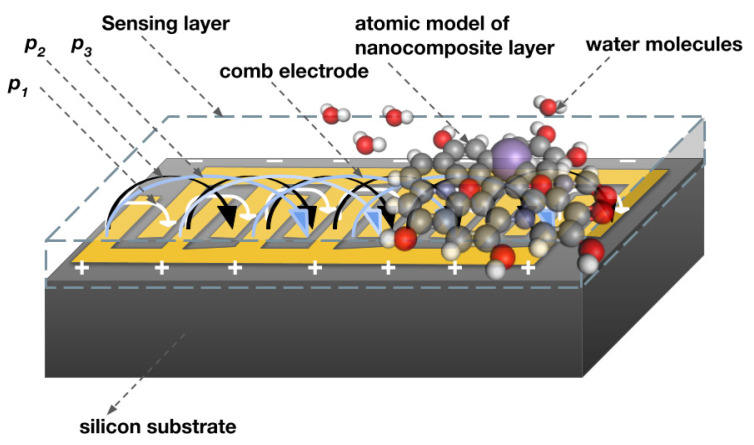
The fringing electric field in IDE of different pitch lengths p_1,_ p_2,_ and p_3_ illustrates the penetration depth over the sensing layer.

As far as in-plane sensing is considered, the fringing capacitance is an important parameter to measure, which is very much dependent on the number of comb fingers and spatial wavelength [35,47]. The fringing electric field between positive and negative electrodes with three different pitch lengths p_1,_ p_2,_ and p_3_ is illustrated in Figure 14. The penetration depth of the electric field is increased by spatial wavelength (λ), but if spatial wavelength increases, the total fringing electric field between consecutive electrodes weakens [44]. This may be the reason for the 28-finger comb electrode having improved sensitivity in terms of capacitance compared to a lesser number of electrode fingers because of the strong accumulation of electric field between consecutive electrodes.

## 5. Conclusions

GO-Zn_1−x_Mn_x_O nanocomposite with facile, single-pot processing procedure and excellent electrical properties for humidity-sensing applications are demonstrated. The functionality of nanocomposite sensing material and sensing platform are orchestrated by theoretical studies and practical experimentation, comparing three materials GO, Zn_1−x_Mn_x_O, and GO-Zn_1−x_Mn_x_O with five different dimensions of comb electrode. An optimized dimension of 6 mm × 3 mm, with 28 fingers, 0.1 mm width, and 0.1 mm spacing reveals the best performance result for simple photolithographic methods (lift-off and metal-etching). The electrode deposition layer is far clear and has fewer defects in the metal etching process. GO-Zn_1−x_Mn_x_O nanocomposite exhibited 82.67 times greater response at 40%RH and 95.7 times more sensitivity at 60%RH. The capacitance as well as resistance sensitivity show excellent consistency and are linear for a wide sensing range of humidity (10%RH–90%RH). GO-Zn_1−x_Mn_x_O nanocomposite also inhibits very high stability, even after months of aging. The response time and recovery time are found to be 4.5 s and 21 s respectively. The device is even tested for hysteresis errors. With these characteristics, the newly developed humidity sensing device exhibited excellent performance in overall aspects. Thus, the GO-based nanocomposite has wonderful potential for future bulk production as an in-plane capacitive as well as resistive humidity sensor for high-precision industries.

## Figures and Tables

**Table 1:** Comparison of different combinations of ide for same dimension of 6 mm × 3 mm sensor.

Number of Electrode Fingers	Finger Width (W) (mm)	Distance between Electrodes (S) (µm)	Spatial Wavelength λ (mm)
10	0.2	200	0.8
14	0.2	200	0.8
16	0.1	100	0.6
20	0.1	100	0.4
28	0.1	100	0.4

**Table 2:** Comparison of effect of number of fingers in comb electrode (n) on total interface capacitance C_t_ (with L = 1.4 mm, W = 0.2 mm for 10 and 14 fingers and L = 1.4 mm, W = 0.1 mm and 16, 20, and 28 fingers, respectively).

Number of Fingers (N)	Cell Factor (*K_cell_*) m^−1^	C_0_ (pF/μm^2^)	C_t_ (pF)
10	54.92	2.76 × 10^−3^	1932
14	38.02	2.85 × 10^−3^	1995
16	32.95	2.878 × 10^−3^	1607
20	26.01	2.917 × 10^−3^	2042
28	18.30	2.96 × 10^−3^	2901

## Data Availability

Not applicable.
